# An Actionable Expert-System Algorithm to Support Nurse-Led Cancer Survivorship Care: Algorithm Development Study

**DOI:** 10.2196/44332

**Published:** 2023-10-04

**Authors:** Kaylen J Pfisterer, Raima Lohani, Elizabeth Janes, Denise Ng, Dan Wang, Denise Bryant-Lukosius, Ricardo Rendon, Alejandro Berlin, Jacqueline Bender, Ian Brown, Andrew Feifer, Geoffrey Gotto, Shumit Saha, Joseph A Cafazzo, Quynh Pham

**Affiliations:** 1 Centre for Digital Therapeutics University Health Network Techna Institute Toronto, ON Canada; 2 Department of Systems Design Engineering University of Waterloo Waterloo, ON Canada; 3 School of Nursing McMaster University Hamilton, ON Canada; 4 Department of Urology Queen Elizabeth II Health Sciences Centre Halifax, ON Canada; 5 Princess Margaret Cancer Centre University Health Network Toronto, ON Canada; 6 Niagara Health System Thorold, ON Canada; 7 Trillium Health Partners Mississauga, ON Canada; 8 Department of Surgery University of Calgary Calgary, AB Canada; 9 Institute of Health Policy, Management, and Evaluation University of Toronto Toronto, ON Canada; 10 Tefler School of Management University of Ottawa Ottawa, ON Canada

**Keywords:** prostate cancer, patient-reported outcomes, nurse-led model of care, expert system, artificial intelligence–powered decision support, digital health, nursing, algorithm development, cancer treatment, AI, survivorship, cancer

## Abstract

**Background:**

Comprehensive models of survivorship care are necessary to improve access to and coordination of care. New models of care provide the opportunity to address the complexity of physical and psychosocial problems and long-term health needs experienced by patients following cancer treatment.

**Objective:**

This paper presents our expert-informed, rules-based survivorship algorithm to build a nurse-led model of survivorship care to support men living with prostate cancer (PCa). The algorithm is called No Evidence of Disease (Ned) and supports timelier decision-making, enhanced safety, and continuity of care.

**Methods:**

An initial rule set was developed and refined through working groups with clinical experts across Canada (eg, nurse experts, physician experts, and scientists; n=20), and patient partners (n=3). Algorithm priorities were defined through a multidisciplinary consensus meeting with clinical nurse specialists, nurse scientists, nurse practitioners, urologic oncologists, urologists, and radiation oncologists (n=17). The system was refined and validated using the nominal group technique.

**Results:**

Four levels of alert classification were established, initiated by responses on the Expanded Prostate Cancer Index Composite for Clinical Practice survey, and mediated by changes in minimal clinically important different alert thresholds, alert history, and clinical urgency with patient autonomy influencing clinical acuity. Patient autonomy was supported through tailored education as a first line of response, and alert escalation depending on a patient-initiated request for a nurse consultation.

**Conclusions:**

The Ned algorithm is positioned to facilitate PCa nurse-led care models with a high nurse-to-patient ratio. This novel expert-informed PCa survivorship care algorithm contains a defined escalation pathway for clinically urgent symptoms while honoring patient preference. Though further validation is required through a pragmatic trial, we anticipate the Ned algorithm will support timelier decision-making and enhance continuity of care through the automation of more frequent automated checkpoints, while empowering patients to self-manage their symptoms more effectively than standard care.

**International Registered Report Identifier (IRRID):**

RR2-10.1136/bmjopen-2020-045806

## Introduction

### Background

Comprehensive models of survivorship care are necessary to improve access to and coordination of care and to address the complexity of physical and psychosocial problems and long-term health needs experienced by patients following cancer treatment [1]. In Canada, for prostate cancer (PCa), follow-up treatment typically consists of specialist visits every 3 to 6 months for the first 5 years, and annually thereafter. During visits, the specialist routinely asks questions about treatment side effects in addition to blood work (ie, prostate-specific antigen [PSA] testing, testosterone). Sometimes imaging tests and or prostate biopsies are completed if PSA values rise [2]. With the increasing demand for posttreatment cancer follow-up care with oncologists at prespecified intervals, clinics are over capacity and lack the ability to prioritize complex patients or address emerging needs [1-3]. There is, therefore, a need for improved sharing of health information, supportive care between oncologist-led follow-up visits, and better care coordination [1,4]. In recent years, nurse-led cancer survivorship models have been widely adopted and accepted as an effective means to support patients at scale; nurse-led survivorship programs in the United States and the United Kingdom have demonstrated clinical effectiveness and cost-effectiveness while yielding high satisfaction among patients and supporting staff [1,5-7]. Systematic reviews suggest better integration of nursing roles in survivorship services will improve the quality of care, patient experience, and health outcomes, and will promote systems-wide cost savings by reducing the need for other health care services [8-11].

### No Evidence of Disease (Ned) Model of Service

In accordance with these best practices, our group conceived the Ned (No Evidence of Disease) nurse-led virtual clinic to support men living with PCa in the posttreatment follow-up phase of their survivorship journey [12]. Patients who have completed treatment and are at low risk of cancer recurrence as determined by their specialist can enroll in Ned clinics. There are two arms of the Ned clinic: (1) baseline Ned Specialist and (2) Ned Nurse.

Ned Specialist contains the usual care touchpoints of the traditional specialist standard of care visits but is conducted asynchronously or with virtual calls when deemed appropriate by the specialist.

Ned Nurse works as an added layer of intervention with patient triaging and decision support guided by the Ned algorithm on top of Ned Specialist. What Ned Nurse and the Ned algorithm add to the Ned model of service is the ability to (1) more frequently monitor the quality of life–related aspects of survivorship care and (2) facilitate more holistic follow-up through the nurse-led service surrounding the algorithm. Within the broader Ned service, Ned nurse coordinators will leverage the embedded algorithm to triage follow-up care for enrolled patients via algorithm-driven tiered alerts that will support the Ned nurse with follow-up care prioritization. Ned Nurse will be embedded within the PCa clinic of each institution from which patients will be recruited. This will allow Ned nurses to liaise with patients’ Ned specialists and follow up with general practitioners as necessary through institution-based processes for interdisciplinary communication. Additionally, the nurse-led model of care provides an opportunity for a high nurse-to-patient ratio, with the intent to support optimized survivorship care and the use of health system resources. Once enrolled, patients are remotely monitored by an advanced practice Ned nurse to identify any deterioration in the quality of life while continuing specialist follow-ups in parallel to assess for cancer recurrence. The assessment schedule is as follows: specialist visits continue to occur at regular intervals (eg, every 6 months, or annually). In preparation for this visit, patients complete a self-report tool like the validated Expanded Prostate Cancer Index Composite for Clinical Practice (EPIC-CP), which has been widely adopted for PCa survivorship symptom monitoring at major cancer centers across Canada [13] and can be completed as needed to monitor outcomes [14]. In between, there are monthly check-ins. The patient is prompted automatically through the system to complete the EPIC-CP. Both providers (specialist and Ned nurse) have shared access to the patient’s medical history, lab results, and self-reported symptoms to maintain continuity of care. However, the Ned nurse is the patient’s primary care provider to resolve their unmet health needs.

### Research Aim and Purpose of the Ned Algorithm

While the application of algorithm-based decision support systems to guide PCa detection and treatment is not new [15-17], previous efforts have not focused on the survivorship context to optimize follow-up care. Therefore, there is an opportunity to develop an expert-informed PCa survivorship care algorithm to support decision-making for both patients and providers. Central to the implementation of the Ned clinics is the ability for nurses to manage the large roster of PCa survivors being followed up in Canada’s largest cancer centers. Focusing these clinics on a stable patient population with “no evidence of disease” increases the feasibility of a high nurse-to-patient ratio to enable efficient and holistic care. The Ned algorithm provides decision support for Ned nurses by automatically triaging patient needs for follow-up (via their EPIC-CP score and change in score), and generates alerts for the nurses to oversee and follow up when necessary in the form of additional support and triaging to appropriate services as needed. Additionally, the Ned algorithm provides direct patient support so that patients may also benefit from tailored guidance to self-manage chronic symptoms [18].

The purpose of this study is to build a novel, rules-based, expert system—the Ned algorithm—for PCa survivors with Ned to support the nurse-led arm of Ned clinics in an ongoing trial [12]. The system consists of relevant survivorship indicators as inputs (EPIC-CP) and outputs actionable insights to a clinician dashboard and patient app. With the assistance of the algorithm, nurses can safely scale their services to remotely monitor patients in the Ned clinic. More importantly, they can perform data-driven and contextualized assessments of which patients require immediate intervention and provide timely care.

## Methods

### Ethics Approval

The process of developing the Ned algorithm was reviewed by the University Health Network (UHN) Quality Improvement Review Committee as part of a quality improvement initiative (QID 20-0114). This algorithm development was part of a larger project to develop and test the Ned platform for which approval was obtained through Clinical Trials Ontario (CTO) with the UHN Research Ethics Board as the board of record (Project ID 3238). This approval is a part of the larger CTO project portfolio, which maintains ethical oversight for all applicable activities associated with the Ned Nurse research program.

### Development of the Ned Rule Set

**Figure 1:**
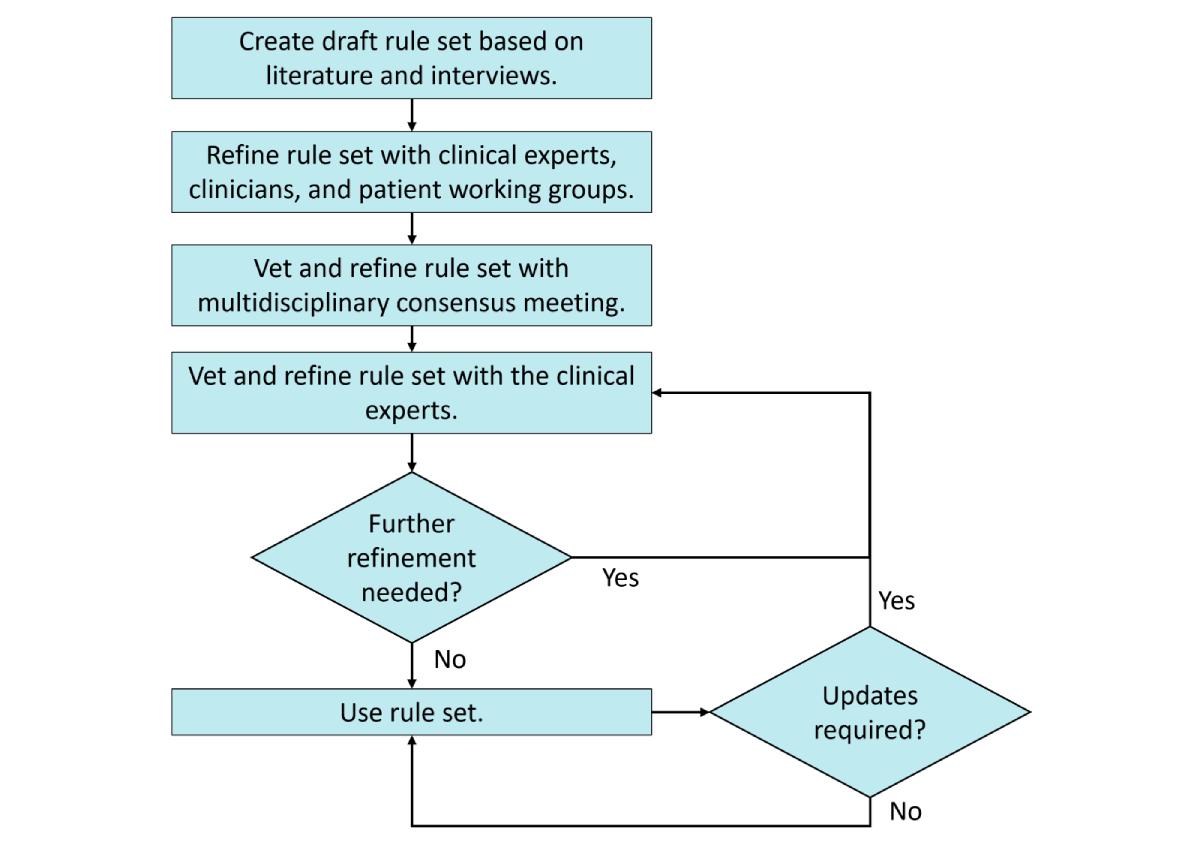
Iterative user-centered process for rule development and validation.

An initial rule set was drafted using results from literature-informed ad-hoc subject matter expert interviews with our clinical champions to validate our foundational understanding of algorithm structure and notation (Figure 1). The draft rule set was then refined through 3 working groups of clinical experts across Canada (eg, nurse experts, physician experts, and scientists; n=20), and patient partners (n=3). The working group discussions were facilitated using the nominal group technique to develop consensus on important PCa survivorship inputs, appropriate care interventions, and system design requirements [19]. Our patient partners provided feedback on the initial lay design of the algorithm, particularly on how to position our survivorship algorithm to more strongly embed patient autonomy and empowerment. The rule set was further refined through a 1-day virtual consensus meeting to validate the proposed symptom inputs and alert outputs. The consensus meeting was recorded and meeting notes were taken for analysis.

### Participants

A purposeful convenience sample was invited to participate in the consensus meeting as voting members based on their clinical and research expertise in oncology and cancer survivorship. Participants were recruited through participating sites for the trial (Ontario, Nova Scotia, and Alberta), as well as from subject matter experts on our investigator team. These participants had specialized expertise in cancer survivorship and PCa survivorship and represented major cancer care centers and research institutions across Canada. Working group participants consisted of clinical experts across Canada (eg, nurse experts, physician experts, and scientists; n=20), and nonvoting patient partners (n=3) to provide expertise on technology development and health service design. Algorithm priorities were defined through a multidisciplinary consensus meeting with clinical nurse specialists, nurse scientists, nurse practitioners, urologic oncologists, urologists, and radiation oncologists (n=17).

### Preparation

We presented content and structure in accordance with a modified protocol from a previous algorithm consensus meeting to develop a heart failure telemonitoring system, which we adapted to meet the specific care needs of the PCa survivorship population [20]. An expert facilitator with experience in leading consensus meetings was recruited to lead the proceedings and support the overall voting process. Prior to the consensus meeting, a detailed package including PCa survivorship literature, a meeting agenda, and potential Ned algorithm inputs and outputs were disseminated to the consensus meeting participants.

### System Refinement and Validation

The consensus meeting used the nominal group technique to develop the decision nodes and pathways of the Ned algorithm [19]. The nominal group technique allows for the pooling of judgment from a group of experts through 2 facilitated rounds of voting. Consensus was defined as at least 75% endorsement from votes. All votes were kept anonymous. In the first round of voting, assent and dissent regarding a particular component of the Ned algorithm (eg, inputs, alert states, and outputs) were assessed using a Zoom (Zoom Video Communications, Inc) poll. Members were asked whether they agreed or disagreed with a particular alert state. Participants who dissented were allocated 30 seconds to share their arguments and opinions with a 60-second response by our team. If consensus was not reached, a second round of voting was initiated. The algorithm alert states all passed the 75% consensus threshold necessary for validation.

### Expert Input Responses and System Refinement

Following the consensus meeting, expert input and responses were used to refine the Ned algorithm and core components. Semistructured interviews (n=10) took place to refine and validate the prototype algorithm with clinical specialists and researchers based on their areas of expertise. Through these interviews, the vetted Ned algorithm, including core survivorship symptom inputs, rules for alert generation, and appropriate survivorship nursing interventions, was identified. Rules for escalation and clinically urgent symptoms were then translated into the algorithm.

### Expert-Informed Validation of Alert State Classification

To validate the refined Ned algorithm, we prepared 2 rounds of fictitious case studies, each comprising 3 unique patients who presented with differing symptoms and needs. These case studies were designed to appraise the Ned algorithm’s ability to discern the unique characteristics of each patient’s reported symptoms, clinical context, the appropriate alert prioritization, and triaging of alerts. The case studies were assessed and analyzed by consensus meeting members (n=25), who were asked to review and prioritize alerts for both case studies and provide roundtable feedback.

## Results

### Algorithm Overview

**Figure 2:**
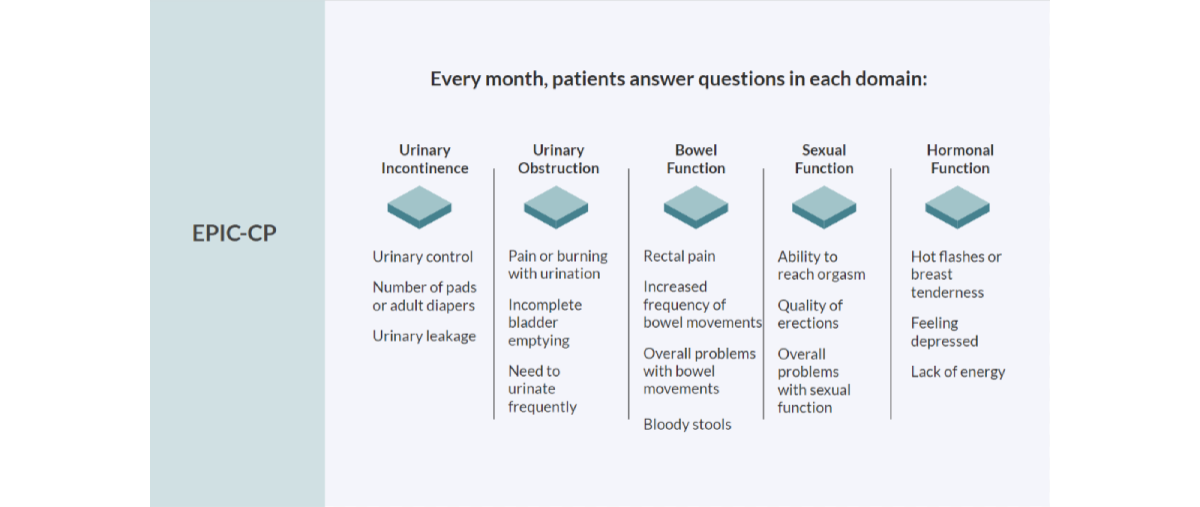
EPIC-CP’s quality of life outcomes are central to the No Evidence of Disease (Ned) algorithm. EPIC-CP is comprised of a 1-page 16-item questionnaire covering questions relating to symptom domains of urinary incontinence (4 questions), urinary obstruction (1 question; 3 subquestions), bowel function (1 question; 3 subquestions), sexual function (3 questions), and hormonal function (1 question; 3 subquestions). Each question is scored categorically from 0, “no problem,” to 4, “big problem,” for a total domain score between 0 and 12 and a total overall prostate cancer quality of life score out of 60 [15]. Additional clinically important, nonscoring questions pertaining to hematuria and bloody stools were added. EPIC-CP: Expanded Prostate Cancer Index Composite for Clinical Practice.

The algorithm takes input from the patient self-report PCa composite index scale (ie, EPIC-CP), and flows to 2 levels of assessment (question and domain levels) to determine 1 of 4 possible triaged alerts (Figure 2). The alerts are green, yellow, orange, and red, where green means all domains have normal status and warmer colors represent higher levels of acuity, with red being the most acute.

### Algorithm Inputs

**Figure 3:**
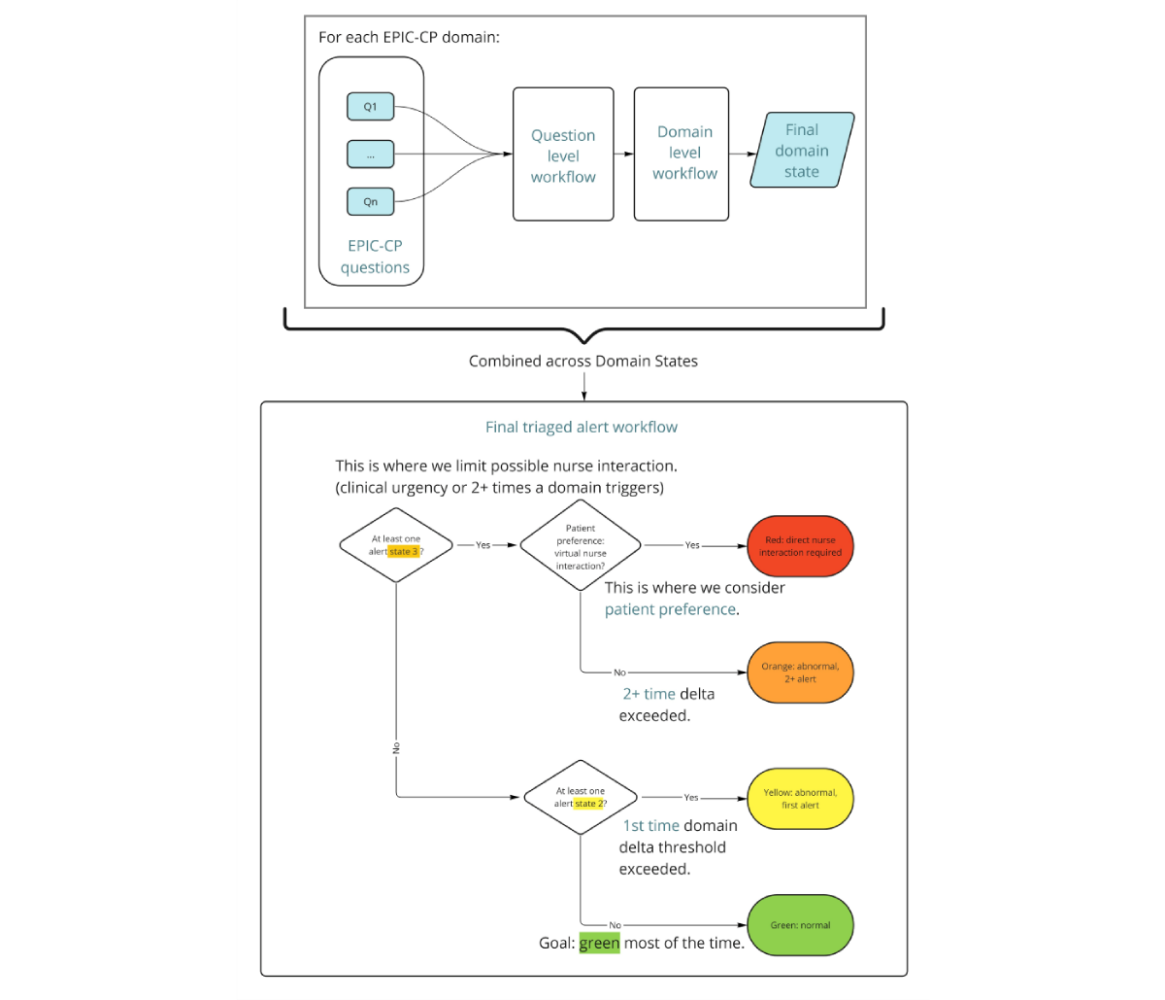
Algorithm overview. Per domain, EPIC-CP questions (Wellness Survey on the patient-facing app) are input into a question-level workflow feeding forward into the domain-level workflow to yield a domain state. Domain states are then combined into a final triaged alert workflow considering clinical urgency, domain state history, domain changes over time (Δ), and patient preference to yield 1 of 4 global alerts: green, yellow, orange, and red. Nurse interaction is required for red alerts, with interaction at their discretion for orange alerts. EPIC-CP: Expanded Prostate Cancer Index Composite for Clinical Practice.

The Ned algorithm ingests health-related quality-of-life outcomes monthly via the EPIC-CP [14]. The EPIC-CP covers 5 domains: urinary incontinence, urinary obstruction, bowel function, sexual function, and hormonal function (Figure 3). Each domain consists of 3 questions scored out of 4, with the domain scored out of 12 and a total score between 0 and 60; higher values mean worse symptoms [14].

### Algorithm Workflows

#### Question-Level Workflow

The question-level workflow is where the response to each EPIC-CP item is analyzed for whether it is a normal response or clinically urgent. Additional details on question-level workflow are available (Multimedia Appendix 1). We determined clinical urgency based on Cancer Care Ontario (CCO) guidelines [21] and consultations with clinical experts. Five clinically urgent items were unanimously validated for clinical appropriateness by our nurse and physician investigators where any patients presenting with these symptoms in the clinic would warrant further investigations. The 5 clinically urgent items are based on differential diagnoses, including (1) pain or burning with urination, (2) weak urine stream or incomplete emptying (urinary tract infection), (3) hematuria, (4) bloody stools (radiation-related side effects), and (5) depression, to determine whether specialized support like counseling, pharmacological treatment (eg, antidepressants), or both of these is warranted. Details of the normal and clinically urgent states are provided in Multimedia Appendix 2.

#### Domain-Level Workflow

The domain-level workflow determines the changes to domain scores over time. We defined 2 Δ (ie, change scores): (1) local Δ to capture changes to scores compared to the previous month and (2) baseline Δ to capture changes to scores compared to a refreshing baseline, where the baseline score gets refreshed every 6 months. To calculate the Δ, patients must have completed at least 2 surveys to calculate a change in score. Where a missing datapoint is present, the algorithm uses the most recent EPIC data compared to the previous month. If the date of completion is outside 3 weeks from the expected due date of the current follow-up month, then this automatically triggers (flagging for patient overdue) a message to the nurse to follow up. The baseline refresh is important to capture large shifts in patients’ trending scores (eg, high score, followed by improvement with slow deterioration). By accounting for both types of Δ, the algorithm is sensitive to capturing rapid (ie, since the previous month) and gradual changes over time (eg, slow-creeping scores). Based on expert consultation with the original authors of the EPIC-CP, we codefined the threshold for both local and baseline Δ domain level states. These thresholds represent minimal clinically important differences (MCID) in scores and can be custom tailored and further informed by literature-defined minimally important differences (MIDs) for the EPIC-26 and EPIC-CP [22,23]. Specific domain thresholds were defined as Δ>0 (ie, urinary incontinence and hormonal function), and Δ>1 (ie, urinary obstruction, bowel function, and sexual function). Clinically urgent questions escalate the state as appropriate (ie, urinary irritation or obstruction, bowel function, and hormonal function). Pragmatically, these thresholds can be tunable based on clinician preference to provide more personalized thresholds that are either more or less sensitive for patients (Multimedia Appendix 1).

Two additional considerations inform the domain-level state. First, a domain-state history is used for pushing appropriate actionable patient-facing feedback to the patient that considers patient symptom chronicity (ie, that the unique “fingerprint” of symptoms for each patient remains fairly stable over time). Second is an escalation based on clinical urgency, which also considers domain-state history. The domain-state outputs are green for normal states and yellow or orange for abnormal states. Yellow is escalated to orange if a Δ threshold is exceeded for the second or more time, or if a clinically urgent symptom is present.

### Algorithm Outputs

After the domain-level workflow, each domain’s state output is combined to provide a global alert in accordance with CCO guidelines and validated through a unanimous expert panel agreement. There are 4 possible alert states (Figure 3):

- Green (normal): This is the alert triggered if no domain state thresholds were met and no support is necessary.
- Yellow (abnormal): This alert is triggered for the first time alerting on a domain, which means the Δ threshold is exceeded for the first time. If this alert is triggered for any subsequent time (ie, second or more times), the alert is escalated to orange.
- Orange (abnormal+): This alert is triggered for the second+ time with elevated domain state (ie, Δ threshold exceeded for a second or more time) or due to a clinically urgent symptom being present. A push from the Ned platform (a mobile patient-facing app) is sent to the patient to ask their preference for a nurse consultation. If the patient indicates they would like a nurse consultation (ie, they accept the interaction), their alert state is updated to red.
- Red (direct nurse interaction required): This alert is triggered if the patient accepts a virtual nurse interaction when one or more orange domain states are present.

When the Ned algorithm outputs any alert, patient-facing actionable feedback is provided as the first line of the response with tailored care steps that outline prescriptive actions patients can enact to self-manage their alerting symptoms. The nurse on the web-based dashboard is shown the patients’ overall alert and summary of domain states and questions factoring into the alert.

## Discussion

### Anticipated Impact of Patient-Facing Actionable Feedback on Patient Self-Care

Given the chronicity of PCa survivorship symptoms, patients living with long-term symptoms may prefer not to receive clinical care despite reporting ongoing symptom experience, especially as symptoms often lack complete resolution [24]. When the Ned algorithm outputs an alert, patient-facing actionable feedback is the first line of response, in the form of domain-specific care steps. This process honors patient autonomy while facilitating a higher patient-to-clinician ratio by providing self-management strategies to minimize the number of required follow-ups. The goal is to provide patients with an arsenal of techniques to self-manage their symptoms as needed. This ensures patients who constantly receive alerts and demonstrate continuous or increasing symptom burden are provided the necessary care while being respectful of their decision to decline direct nursing intervention. To ground the algorithm outputs as a tractable example, a fictitious patient case study is outlined at baseline and 2 timepoints (Multimedia Appendix 3).

We anticipate the successful application of this survivorship algorithm will support the delivery of holistic nurse-led care and facilitate improved quality of life and survivorship experience with a high patient-to-nurse ratio. Our approach provides patient-facing actionable feedback on patient self-care to promote independent access to self-care education without necessarily having to see a care provider.

People who have been having prostate cancer treatment symptoms for a long time are familiar with how to manage it...they might not want medical advice because they know they can manage it and that there is no true solution.

Patient A

### Overcoming Ned Algorithm Operationalization Barriers

Our intent was to incorporate the Ned algorithm into our Ned virtual clinics to improve efficiencies while enhancing the quality of care (eg, empower patients with the ability to self-manage symptoms and aspects of their cancer care). To operationalize the Ned algorithm there are 3 additional considerations. First, within the context of nurse-led care provision, the Ned algorithm must have a clearly defined scope in terms of eligibility and appropriate use. Second, robust provider education is required to ensure appropriate clinical application and utility. To this end, we engaged senior nurse experts in oncology care to inform the creation of a nursing curriculum that will position Ned nurses to understand both algorithm-related and non–algorithm-related core care intervention pathways. Third, a formal evaluation is warranted of care provider responses to algorithm alerts and Ned as clinical decision support.

### Future Directions

#### The Challenge and Opportunity to Advance the Ned Algorithm With PSA

While it is difficult to obtain unanimous agreement on PSA and what constitutes recurrence, incorporating PSA may be the most obvious algorithm feature as a first screening step for future directions of the Ned algorithm. For example, based on a recent systematic review, most publications use a PSA of greater than 0.4 ng/mL [25] while the RTOG-ASTRO (Radiation Therapy Oncology Group–American Society for Therapeutic Radiology and Oncology) Phoenix Consensus Conference uses a definition of a PSA increase of more than 2 ng/mL regardless of the nadir value [26], and in Canada, it has been recommended to use a combination of testosterone and PSA levels (≤0.7 nmol/L and ≤2 ng/mL, respectively) [27]. Others have proposed age-specific thresholds [28] and the European Association of Urology (EAU) Prostate Cancer Guidelines Panel’s recommendations state that “The indication for further treatments should not be based on meeting a threshold PSA recurrence as defined above alone, but should depend on an individualized risk for progression.” [25]. Especially with these nuances, there exists an element of subjectivity for clinicians assigning a clinical stage with errors in the clinical stage assignment of greater than 35% [29]. We report here on a more conservative algorithm. As part of future work, we aim to marry these 2 approaches through a data-driven clinical support system to facilitate clinician trust while reducing data fatigue.

#### Refining Algorithm Thresholds for a More Flexible Response

The focus of this project was to develop the algorithm. Evaluation of the algorithm’s pragmatic feasibility to support a high patient-to-nurse ratio, and further validation of algorithm output compared to a human assessor is part of ongoing work. While we based the thresholds on expert recommendations, practically, there may be more value in tailoring thresholds for each patient through an initial consultation with a Ned nurse. We also understand each patient will have their own unique “fingerprint” of side effects following treatment. Currently what will be trialed are the thresholds as defined in the manuscript. However, to address potential oversensitivity for each patient’s unique baseline values, in consultation with our clinical partners, we discussed fine-tuning or updating the thresholds on a case-by-case basis to prevent alert fatigue both for patients and the Ned nurse. However careful consideration is necessary to mitigate the risk of potential maladaptation of the algorithm. Additionally, granularity beyond the first and subsequent alerts may be more effective to assist patients with self-resolving their symptoms. Continuing with the evaluation of this research will help refine and optimize both thresholds and patient-facing resources.

### Limitations and Next Steps

Patient user feedback was obtained from members of our patient partner council, who are very active in their care and therefore may not represent patients who play a less active role in their care. Future directions should include additional input and perspectives from more diverse patient expertise to further corroborate these findings. In line with conventional user-centered design, a summative pragmatic pilot evaluation is needed to ensure the usability of the algorithm prior to mass deployment to ensure no issues could produce adverse events. As part of the next steps, we will evaluate the incorporation of the Ned algorithm’s prescriptive patient-facing actionable feedback on the patient experience, perceptions of patient empowerment, clinical outcomes and clinical efficacy, degree of expected missing data, and missing-data mitigation strategies.

### Conclusions

The Ned algorithm is positioned to facilitate PCa nurse-led care models with a high nurse-to-patient ratio. This novel expert-informed PCa survivorship care algorithm contains a defined escalation pathway for clinically urgent symptoms while honoring patient preference. Though further validation is required through a pragmatic trial, we anticipate the Ned algorithm will support a high patient-to-nurse ratio and enhanced efficiency with empowering patients to self-resolve their symptoms and improve their quality of life.

## Appendix

Multimedia Appendix 1 Workflows and domain state history. This multimedia appendix provides supplementary information regarding the question and domain-level workflows and domain state history including in-depth algorithm workflow diagrams (Figures S1 and S2).

Multimedia Appendix 2 Considering clinical acuity. This multimedia appendix provides supplementary information regarding clinical acuity and how this is captured for standard and special domains. It includes in-depth algorithm workflow diagrams for urinary incontinence (Figure S3), bowel function (Figure S4), and hormonal function (Figure S5).

Multimedia Appendix 3 Fictitious case study. This multimedia appendix provides a fictitious patient case study to illustrate how the No Evidence of Disease (Ned) algorithm flows from scenario to output at baseline and two follow-up time points (Table S1).

## Abbreviations

ASTRO: American Society for Therapeutic Radiology and Oncology
CCO: Cancer Care Ontario
CTO: Clinical Trials Ontario
EAU: European Association of Urology
EPIC-CP: Expanded Prostate Cancer Index Composite for Clinical Practice
MCID: minimal clinically important differences
MID: minimally important difference
Ned: no evidence of disease
PCa: prostate cancer
PSA: prostate-specific antigen
RTOG: Radiation Therapy Oncology Group
UHN: University Health Network

## Data Availability

Data sharing is not applicable to this paper as no data sets were generated or analyzed during this study.
