# Supplementary material for: An Actionable Expert-System Algorithm to Support Nurse-Led Cancer Survivorship Care: Algorithm Development Study
Source: JMIR Cancer. 2023 Oct 4;9:e44332. doi: 10.2196/44332 (PMC10585445; doi:10.2196/44332)
Supplement: Multimedia Appendix 1 [file cancer_v9i1e44332_app1.docx]

# Multimedia Appendix 1: Question and Domain Level Workflows, and Domain State History

For the question-level workflow, questions from the Expanded Prostate Cancer Index Composite for Clinical Practice (EPIC-CP) survey are assessed for their clinical urgency (Figure S1), where clinically urgent symptoms trigger an abnormal question state considered in the domain-level workflow (Figure S2). The domain-level workflow also considers the patient’s state history for each domain to account for patients having their own unique fingerprint of chronic symptoms due to survivorship symptom chronicity. For example, a patient alerting for the first time on an EPIC-CP domain may require different symptom management strategies compared to a patient alerting for the second or third consecutive month. For this reason, we consider their first EPIC-CP as a baseline to which the algorithm assesses for meaningful changes from each patient’s baseline. Compared to the baseline, the number of state occurrences for a given domain or special question is used as counters to determine which set of educational resources should be provided to the patient.

Specifically, 2 Δ are calculated, the first is a local Δ relative to the previous month to capture local changes, and the second is a baseline Δ relative to the baseline assessment to capture any changes relative to baseline in symptomatology. This accounts for cases where month-to-month a patient’s symptom severity slightly worsens but not enough to trigger an alert from the local Δ. The first time a domain Δ meets a threshold, a Yellow, domain state is generated. The second or subsequent time a domain threshold is met, an Orange clinically urgent state is generated which has implications around patient preference. For example, if after a month of trying one care step, a domain score continues to worsen, a new care step will be provided. Pragmatically, this implies that unless a patient experienced any degree (>0) of a clinically urgent symptom, the highest first-time state generated is Yellow with the only possible outcome of providing educational resources for self-management. In the former case, the Orange state is triggered where, in addition to care steps provided, the patient is provided with an opportunity to either continue self-managing or have a virtual nurse consult.

**Figure S1.** Question level workflow to assess whether there was an abnormal response on each question to trigger a Yellow state.

*
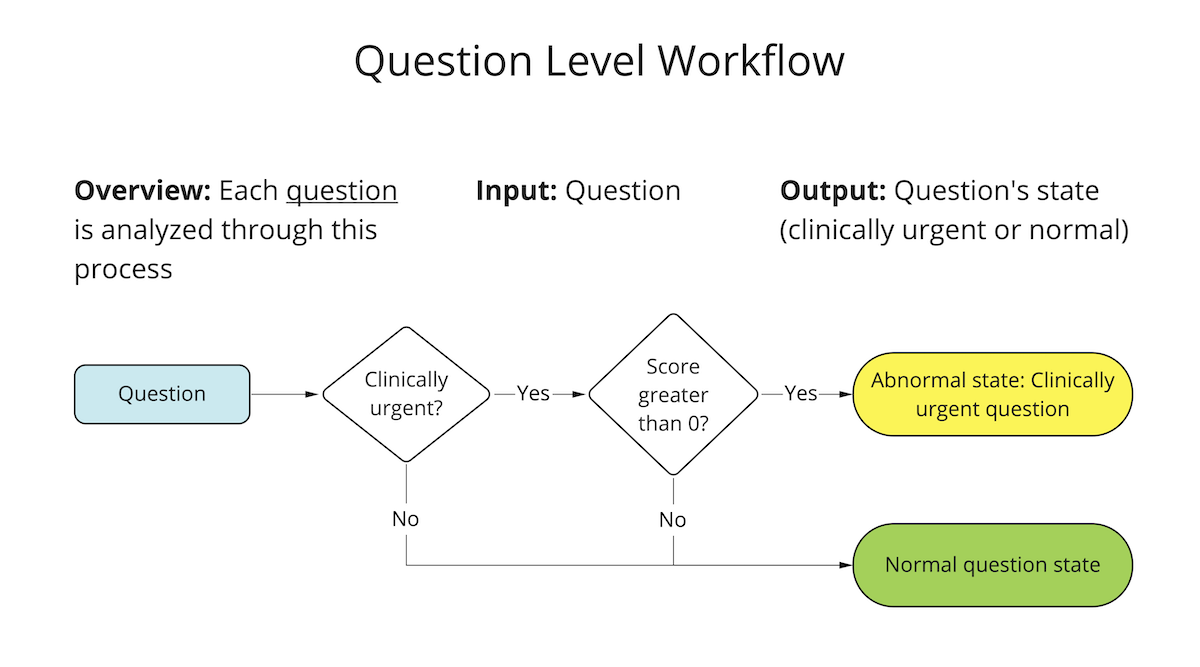
*

**Figure S2**. Domain-level workflow to assess whether an alert should be escalated based on clinical urgency (orange), or based on either a local or baseline Δ across the domain (yellow).

*
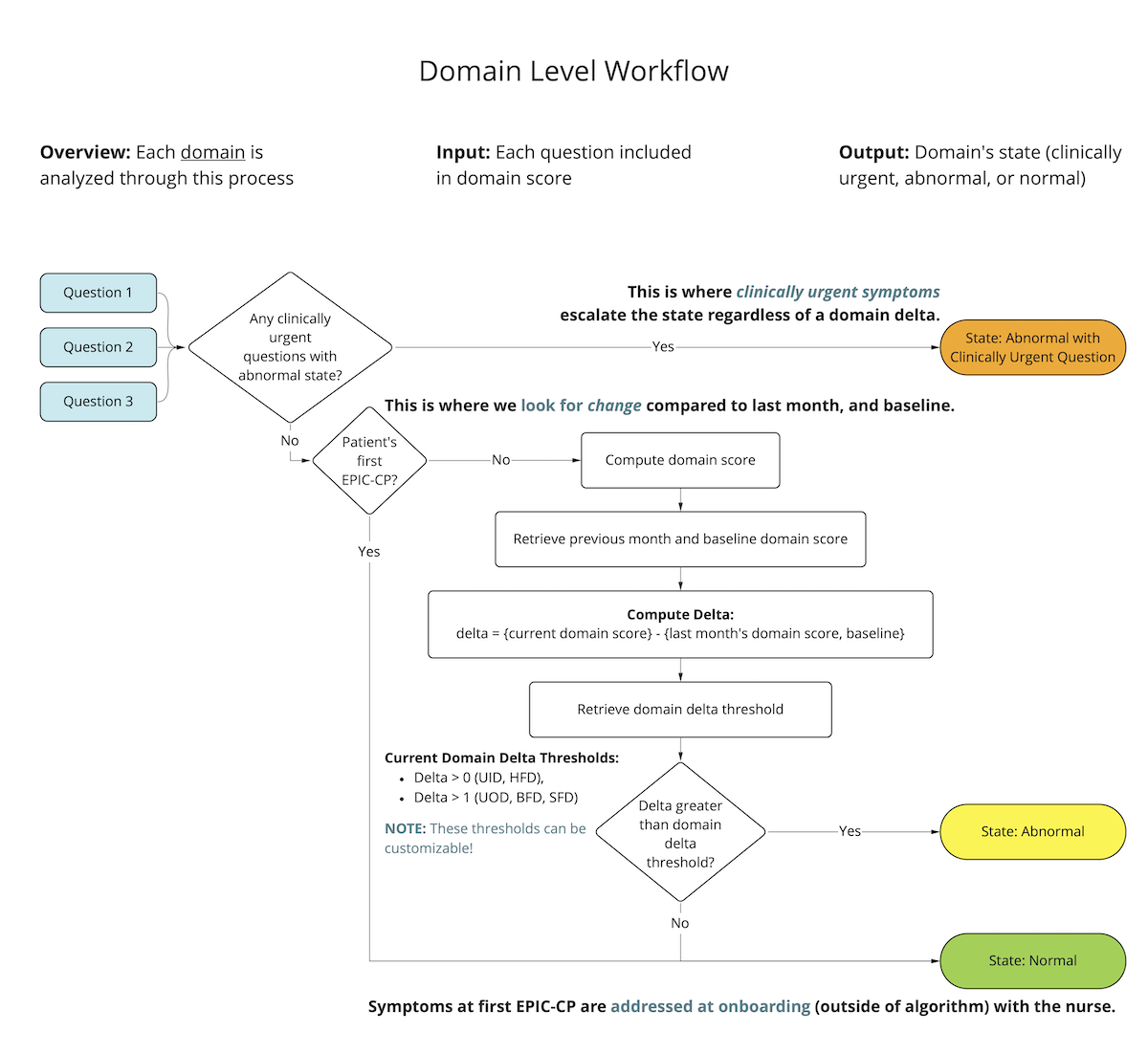
*
