# Supplementary material for: An Actionable Expert-System Algorithm to Support Nurse-Led Cancer Survivorship Care: Algorithm Development Study
Source: JMIR Cancer. 2023 Oct 4;9:e44332. doi: 10.2196/44332 (PMC10585445; doi:10.2196/44332)
Supplement: Multimedia Appendix 2 [file cancer_v9i1e44332_app2.docx]

# Multimedia Appendix 2: Considering Clinical Acuity

The general format of standard domain questions flows from question level to domain level assessment (Figure S3). For standard domains, for a given domain, care steps are provided based on the number of occurrences of abnormal responses above a threshold. This ensures that patients are provided with a fresh set of self-management resources at each timepoint to support the patient in self-resolving their symptoms. The difference between standard domain workflow and domains with special questions arises for 2 types of special cases: one for bowel function and urinary obstruction, and one for hormonal function which are described next.

The first type of special case domain is for bowel function (and urinary obstruction). For this type of special case domain, the nonscoring item pertaining to bloody stools/hematuria, informed by our expert panel, is a clinically urgent symptom with the need to assess etiology. As such, we see that item Q6(d), regarding bloody stools, appropriately does not inform the domain level workflow since it is a nonscoring item for the Expanded Prostate Cancer Index Composite for Clinical Practice (EPIC-CP) (Figure S4). Instead, it provides a parallel pathway for triggering the bowel function domain whenever bloody stools may be experienced (ie, a question threshold >0). This is a poignant difference from the standard domains as it also allows for a parallel escalation pathway for direct nurse consultation based on this one separate item. In terms of discerning which care steps are provided, the number of occurrences for the bowel function domain is compared against the bloody stool trigger state. The maximum number of occurrences is what dictates which resources get shared to prevent duplication or skipped care steps. Hematuria is treated in an equivalent fashion; it does not contribute to scoring, it triggers a separate escalation pathway, and history for the domain and nonscoring item are considered together to determine which care step is provided.

The second type of special case domain is for hormonal function. For this domain, question Q10(b) pertains to feelings of depression. While hormones can govern mental health and wellbeing, care steps to address this clinically urgent symptom require different tacts than other symptoms pertaining to hormonal function like breast tenderness or hot flashes. As such, we see that not only does this question inform the domain-level assessment, but the no evidence of disease (Ned) algorithm also contains a parallel pathway to provide depression-specific resources (Figure S5). This means that if the hormonal domain triggers when a patient is experiencing any symptoms of depression, they will receive both depression-specific and hormonal function domain care steps. In contrast to the instance of bloody stools and hematuria, since the depression question also informs the domain-level assessment, there was no requirement for a separate escalation pathway. Instead, the ability for escalation from clinical urgency is captured with the previously described domain-level workflow (ie, escalating to an Orange state if there is any degree of blood in stool or urine).

**Figure S3**. Standard domain flow. Here we see the standard flow of urinary incontinence. The flow for the sexual function domain is the same.

*
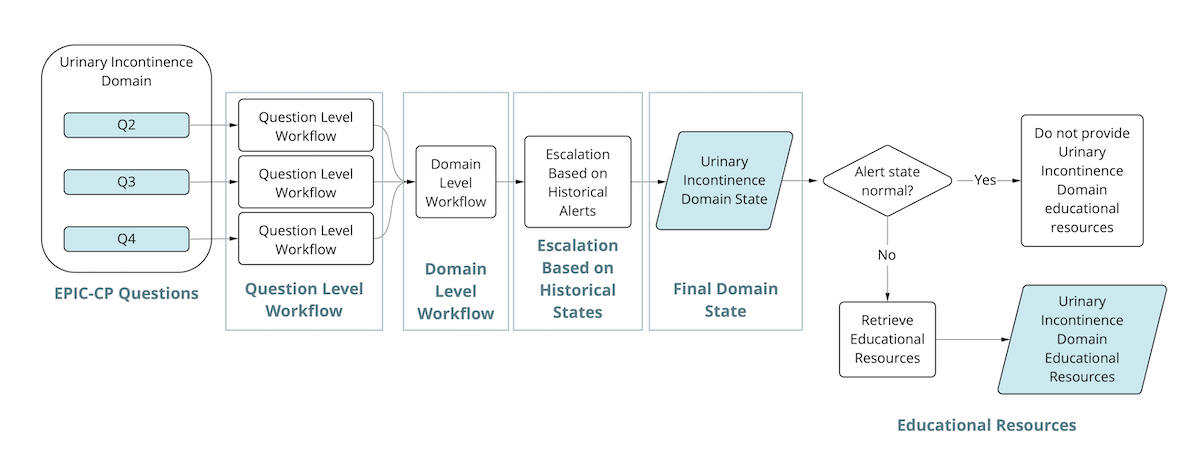
*

**Figure S4**. The “special case domain” flows for bowel function including a separate pathway for the clinically urgent “bloody stool” symptom. Urinary obstruction follows the same flow as hematuria. Both have parallel pathways allowing for escalation with the opportunity for a nurse consult or symptom-specific care steps (hormonal function, depression).

*
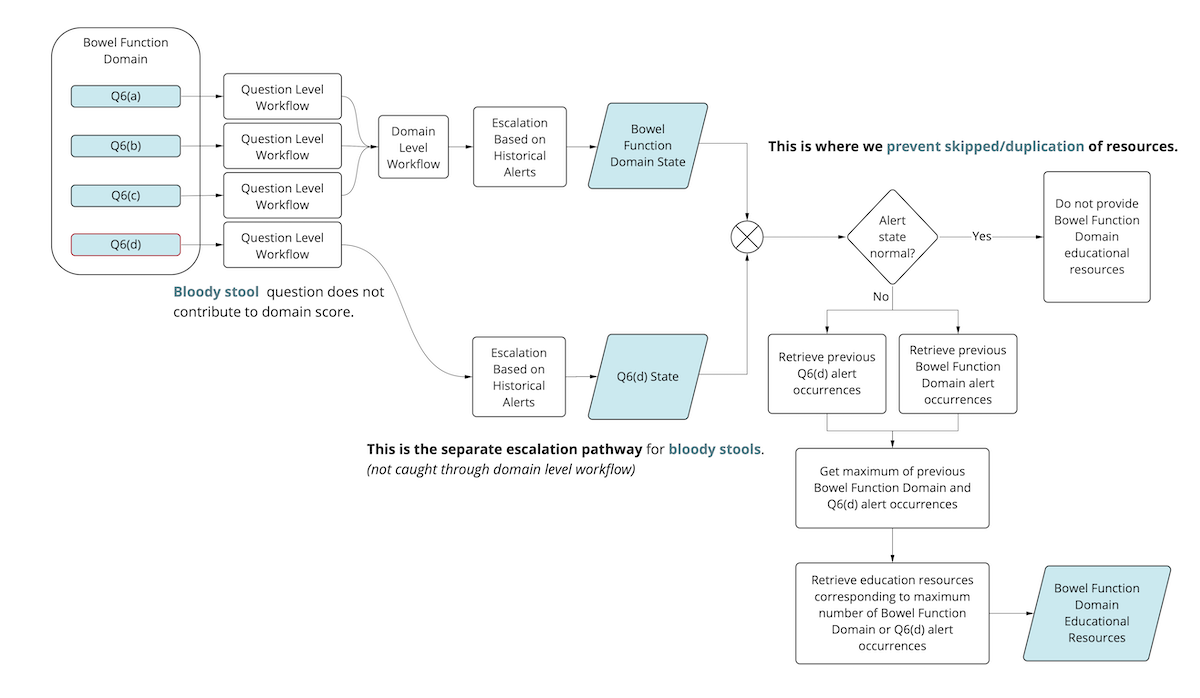
*

**Figure S5**. The “special case domain” flows for hormonal function for the clinically urgent “depressive” symptom on question Q10(b). This special case domain flow contains parallel pathways allowing for symptom-specific care steps (hormonal function, depression) in addition to domain-specific hormonal function care steps.

*
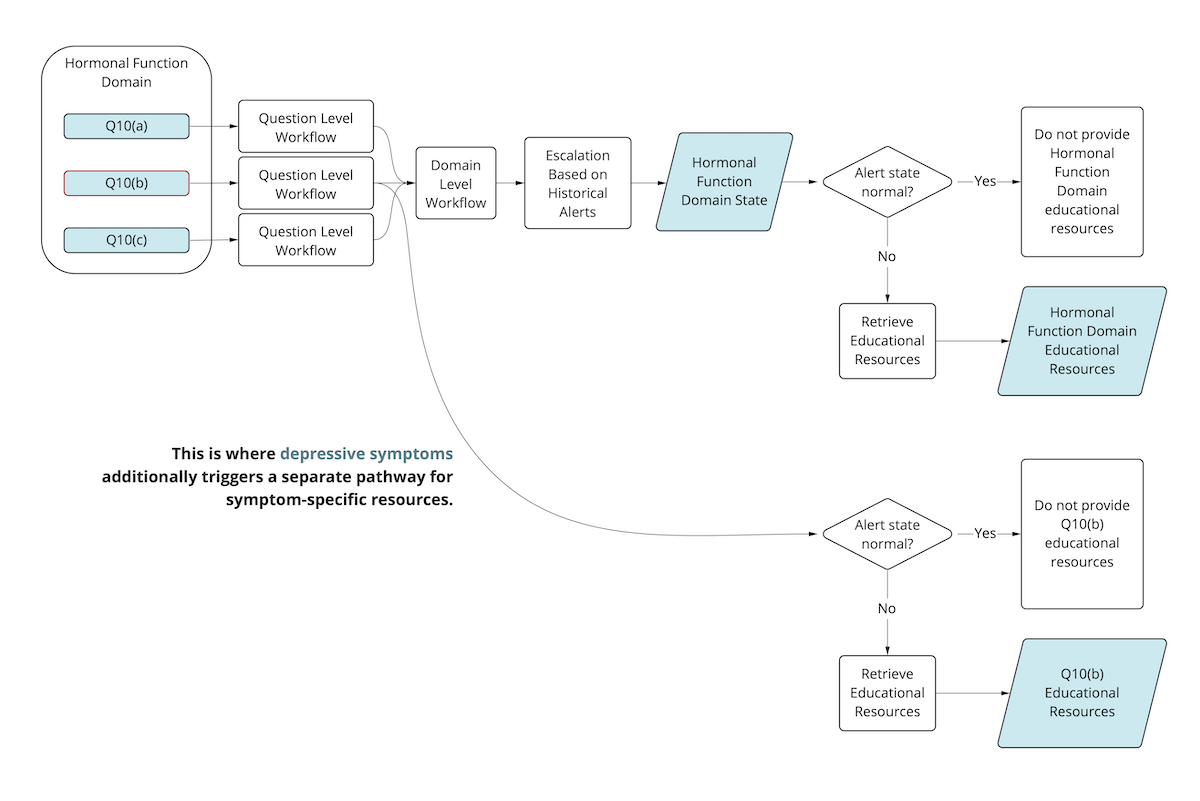
*
