# Supplementary material for: An Actionable Expert-System Algorithm to Support Nurse-Led Cancer Survivorship Care: Algorithm Development Study
Source: JMIR Cancer. 2023 Oct 4;9:e44332. doi: 10.2196/44332 (PMC10585445; doi:10.2196/44332)
Supplement: Multimedia Appendix 3 [file cancer_v9i1e44332_app3.docx]

# Multimedia Appendix 3: Fictitious Patient Case Study

#### The following description provides an overview of how the no evidence of disease (Ned) algorithm works through the worked example with flow from scenario to output (Table S1).

#### Time Point 0: Baseline Assessment

John Smith completes his monthly Expanded Prostate Cancer Index Composite for Clinical Practice (EPIC-CP) symptom domain questions at baseline and he notes that he is experiencing problems with urinary obstruction. Since it is his baseline EPIC-CP, as part of his onboarding, a nurse will provide him with appropriate educational resources to self-manage for that first month. This defines his personalized baseline.

#### Time Point 1: One Month After Baseline

One month later, while John is still experiencing urinary obstruction symptoms, they have not worsened so no abnormal states are generated from this symptom domain. New to this time point are symptoms pertaining to hormonal functioning: lack of energy, and feelings of depression. His total domain score compared to last month (also baseline) yields a hormonal function domain Δ of +5. This is above the threshold of a minimal clinically important difference (MCID) of 1 or greater and is flagged as an abnormal state. While it is his first abnormal state in this domain (Yellow state), the state contains a clinically urgent symptom (depression) and escalates to an Orange state triggering the direct virtual nurse consultation offer. John declines so the final global algorithm output is: Orange alert. John receives domain and depressive symptom-specific care steps.

#### Time Point 2: Two Months After Baseline

At the second time point, John’s urinary obstruction symptoms remain stable (ie, not worsening) so his domain Δ is below the abnormal state trigger threshold. His depressive symptoms and lack of energy symptoms resolved or stayed constant keeping his Δ for hormonal function below the abnormal state trigger threshold. New to this time point, John experienced increased bowel movement frequency and had a bloody stool. His bowel domain score total was 1 out of 4 from the increased frequency (blood stool is nonscoring). The bowel function domain MCID Δ was greater than 1 so it does not trigger a bowel function domain abnormal state. If he had scored 2 out of 4 for increased bowel frequency, it would have triggered a yellow state (first-time alerting on the domain). However, what supersedes the bowel domain score was the bloody stool symptom independently triggering an Orange state. When prompted with the opportunity to consult with a nurse, he accepts. In addition to being provided with a domain-level care step, he receives a virtual consult with the Ned nurse.

**Table S1.** John Smith: Illustrating no evidence of disease’s (Ned’s) stepped approach for patient symptom self-management.

| Criteria | T0: Baseline | T1: Baseline + 1 mo^a^ | T2: Baseline + 2 mo |
| --- | --- | --- | --- |
| Scenario | The EPIC-CP^b^ is completed at baseline to determine subsequent local changes (local Δ) and changes from baseline (baseline Δ). | John is experiencing reduced urinary obstruction symptoms. He is also experiencing depressive symptoms and a lack of energy. | John notices blood in his stool and is concerned. His UO^c^ symptoms are stable. His HF^d^ symptoms have subsided. |
| Symptoms experienced: EPIC-CP “Wellness Survey” responses | Baseline symptoms (UO)  Pain or burning*:* 3/4  Urinary frequency*:* 2/4.  TDS^e^ UO: 5/12  “John scores 0 on all other questions.” | New symptoms (HF)  Feeling depressed: 2/4  Lack of energy: 3/4  TDS UO: 4/12  Baseline Δ: –1; NO alert triggered  TDS HF: 5/12  Δ: +5; domain triggered (1st time) | New symptoms (BF^f^)  Increased frequency: 1/4  Bloody stool: 1/4  TDS BF: 1/12  Baseline Δ: +1; NO alert triggered (Δ< threshold of >1 for BF domain) |
| Urgent follow-up symptoms | Regular Domain (CUS^g^) Pain or burning with urination, urinary frequency (first-time). | Special Case (CUS) Depressive symptom; Domain triggers alert (first time), additional symptom-specific care step provided (first time). | Special Case (CUS) Bloody stool; question directly triggers an alert |
| Global algorithm alert state output | This is the baseline survey. | Orange Alert: Yellow state triggered by hormonal function domain. The CUS supersedes and escalates to Orange state prompting an opportunity for direct nurse consultation. John declines remaining at an Orange alert. | Red Alert: Orange state was triggered by a question directly prompting an opportunity for direct nurse consultation. John accepts and the Orange alert is escalated to a Red alert. |
| Intervention | At boarding, the nurse works directly with John to provide symptom management resources. | HF domain care step provided. Depression-specific care steps provided. | BF domain care step provided. Direct Nurse Interaction Required. |

^a^mo: month.

^b^EPIC-CP: Expanded Prostate Cancer Index Composite for Clinical Practice.

^c^UO: urinary obstruction.

^d^HF: hormonal function.

^e^TDS: total domain score.

^f^BF: bowel function.

^g^CUS: clinically urgent symptom.
